# P117: Hand hygiene compliance in intensive care units in the Madrid region

**DOI:** 10.1186/2047-2994-2-S1-P117

**Published:** 2013-06-20

**Authors:** M Cantero Caballero, P Rodriguez, R Pla, AB Jimenez, A Sanchez, C Rodriguez

**Affiliations:** 1 Department of Preventive Medicine and Quality Management, General University Hospital Gregorio Marañón, Madrid, Spain

## Introduction

HH compliance rates are universally low, leading to unacceptably high rates of healthcare associated infections (HAI). Sice 2010, the Madrid Regional Healthcare System has implemented a regional strategy to improve HH compliance, following the WHO multimodal strategy. HAI rates are especially high in intensive care units (ICU); therefore an effort should be made to evaluate the HH compliance in these units.

## Objectives

The aim of our study was to evaluate the HH compliance in ICUs in the Madrid Region after the implementation of a multimodal strategy.

## Methods

Direct observation according to the WHO observation method was performed before and after the implementation of the multimodal strategy in 22 medical ICUs belonging to the hospitals of the Madrid Regional Healthcare System during March and April 2010 and 2011 by previously trained and evaluated health care professionals.

## Results

We observed 1413 HH opportunities in 2010 and 1614 in 2011. HH compliance was 36,9% (95%CI: 34,4-39,4) in 2010 and 45,11% (95%CI: 42,7-47,5) in 2011. HH compliance rates according to My five moments for hand hygiene in 2010/2011 were: 1.Before touching a patient 27,1/38,7%; 2.Before clean/aseptic procedure: 16,3%/21,9%; 3.After body fluid exposure risk: 40,0%/52,0%; 4.After touching a patient: 57,9%/64,4%; 5.After touching patient surroundings: 29,8%/29,0%. When analyzed by professional category, nurses achieved the highest HH compliance rates (43,3%/47,6% ) while physician residents ranked the worst (18,2%/15,2%) in the 2 years of observation. Alcohol-based solutions were used in 55,7% of the opportunities in 2010 and in 58,0% in 2011.

## Conclusion

Despite the fact that HH compliance improvement was statistically significant after the implementation of a multimodal strategy, acceptable HH compliance remains an objective yet to be achieved in ICUs where its compliance should be a must. Lack of hand hygiene is highly worrisome in the “before moments” and in physician residents, and interventions should target these problems.

## Disclosure of interest

None declared

